# Discovery of potential ALK inhibitors by virtual screening approach

**DOI:** 10.1007/s13205-015-0336-z

**Published:** 2016-01-09

**Authors:** Anish Kumar, V. Shanthi, K. Ramanathan

**Affiliations:** Industrial Biotechnology Division, School of Bio Sciences and Technology, VIT University, Vellore, Tamil Nadu 632014 India

**Keywords:** Non-small cell lung cancer, Crizotinib, Mutation, Virtual screening, Molecular docking, Molecular dynamic simulation

## Abstract

Crizotinib is an anticancer drug used for the treatment of non-small cell lung cancer. Evidences available suggest that there is a development of an acquired resistance against crizotinib action due to the emergence of several mutations in the ALK gene. It is therefore necessary to develop potent anti-cancer drugs for the treatment of crizotinib resistance non-small cell lung cancer types. In the present study, a novel class of lead molecule was identified using virtual screening, molecular docking and molecular dynamic approach. The virtual screening analysis was done using PubChem database by employing crizotinib as query and the data reduction was carried out by using molecular docking techniques. The bioavailability of the lead compounds was examined with the help of Lipinski rule of five. The screened lead molecules were analyzed for toxicity profiles, drug-likeness and other physico-chemical properties of drugs by OSIRIS program. Finally, molecular dynamics simulation was also performed to validate the binding property of the lead compound. Our analysis clearly indicates that CID 11562217, a nitrile containing compound (pyrazole-substituted aminoheteroaryl), could be the potential ALK inhibitor certainly helpful to overcome the drug resistance in non-small cell lung cancer.

## Introduction

Lung cancer is the prominent cause of cancer deaths in the world and a global issue to be addressed (Siegel et al. 2012). Lung cancer is broadly classified into two main types based upon their histology, which are non-small cell lung cancer (NSCLC) and small cell lung cancer (SCLC). The most common forms of NSCLC are adenocarcinoma (ADC) and squamous cell carcinoma (SCC) (Skarda et al. 2008). Chromosomal rearrangements in the anaplastic lymphoma kinase (ALK) gene that codes for anaplastic lymphoma kinase has been identified as one of the causes of NSCLC. There are two types of tyrosine kinase, receptor and cytoplasmic tyrosine kinase. The ALK is a cytoplasmic tyrosine kinase where crizotinib (a potential anticancer drug used in the treatment of NSCLC) is bound. Chromosomal rearrangements involving the ALK gene occur in different malignant conditions, including anaplastic large cell lymphoma (ALCL) and inflammatory myofibroblastic tumor (IMT) (Chiarle et al. 2008). These rearrangements lead to the expression of ALK fusion genes. ALK fusion gene possesses different properties from the two genes that it was originally derived from, can then code for the new ALK fusion protein, which is abnormally and constitutively activated. The new protein contains the tyrosine kinase domain of ALK and the coiled coil domain of EML4. The coiled coil domain of EML4 allows this protein to bind with other ALK fusion proteins and form dimerised and activated fusion proteins (Katayama et al. 2012). The most prevalent ALK fusion oncogene in NSCLC is the echinoderm microtubule-associated protein-like 4 (EML4)–ALK fusion gene and is present in 4–5 % of cases of NSCLC (Young et al. 2010). An inversion in the chromosome 2 brings together the 5′ end of the EML4 gene and the 3′ end of the ALK gene resulting in the formation of the EML4-ALK fusion gene (Shaw and Solomon 2011). The affected person tend to have typical clinical features like early age of onset, little or absence of any smoking history (Shaw et al. 2009). Some of the drugs commonly used for the chemotherapeutic treatment of lung cancer are Bevacizumab, Carboplatin, Cisplatin, Crizotinib, Docetaxel, Erlotinib, Etoposide, Gemcitabine, Irinotecan, Paclitaxel, Pemetrexed, and Vinorelbine. Targeted drug therapy is used against NSCLC of which tyrosine kinase inhibitors are amongst the best method in treatment methodology. In particular, crizotinib is one such tyrosine kinase inhibitor which is the first drug to have gained FDA approval for the treatment of NSCLC in 2011 (Ou, 2011). Although crizotinib has proved itself as an efficient counter to ALK type NSCLC, acquired resistance has made its beneficial effects temporary and has emerged as a major roadblock for crizotinib. The literature evidences available indicates that L1196M (the “gatekeeper” mutation) and G1269A are the two most commonly found secondary mutations in the *ALK* kinase domain. In a few cases, patient harbored with both mutation (Kim et al. 2013). Of note, the available evidence indicates that ALK double mutation (L1196M, G1269A) is one of the main causes for crizotinib resistance (Doebele et al. 2012; Molina et al. 2008). The prevalence of ALK double mutation (L1196M, G1269A) is also significantly higher than other mutation. These situations urge the development of new and more effective ALK inhibitors especially for the treatment of drug resistance NSCLC. For years, computational techniques in particular virtual screening (VS) have proven to be of great use to make the drug development process faster and less expensive. The available literature evidences also suggested that VS techniques proved to be efficacious in making qualitative predictions that discriminated active from inactive compounds (Oprea 2000; Chen 2008). Therefore, in the present investigation, we have employed VS technique to address the crizotinib resistance in NSCLC. We hope that this approach certainly helpful for the experimental biologist to figure out the potent candidates for NSCLC.

## Materials and methods

### Data set

The three-dimensional (3D) structure of native and mutant (L1196M, G1269A) ALK structures were retrieved from the crystal structures of the Brookhaven Protein Data Bank (PDB) for the analysis (Berman et al. 2000). The corresponding PDB codes were 2XP2 and 4ANS for the native and mutant structures, respectively (Cui et al. 2011). Crizotinib was used as the small molecule for our study. The SMILES strings of the crizotinib and the lead molecules were collected from PubChem (Feldman et al. 2006) and submitted to CORINA for constructing the 3D structure of molecule (Gasteiger et al. 1990). The 3D structure of target proteins (2XP2 and 4ANS) drug molecule and lead compounds was energy-minimized using GROMACS package 4.5.3 adopting the GROMOS43a1 force field parameters before performing the computational analysis (Hess et al. 2008; Spoel et al. 2005).

### Virtual screening

Virtual Screening (Shoichet 2004) is an important technique in computer-assisted drug discovery for screening of potential molecule from the database. This approach becomes popular in the pharmaceutical research for lead identification. Diminution of the massive virtual chemical space of small organic molecules and to screen against a specific target protein is the basic goal of the virtual screening (Tondi et al. 1999). In the present study,

virtual screening technique performed with the help of PubChem database by employing crizotinib as a query (Bolton et al. 2008). It is worth stressing that PubChem database holds over 27 million records of unique chemical structures of compounds (CID) derived from nearly 70 million substance depositions (SID). The publicly available PubChem database provides great opportunities for scientists to perform VS process (Xie 2010). Several hits were obtained from the PubChem database, which were further analyzed using molecular docking studies.

### ADME and toxicity

The bioavailability of the lead compounds was examined with the help of Lipinski’s rule of five (Lipinski et al. 1997). The molecular properties such as logP (partition coefficient), molecular weight (MW), or counts of hydrogen bond acceptors and donors in a molecule were utilized in formulating ‘‘rule of five’’ (Ertl et al. 2000). The rule states that most molecules with good membrane permeability should have molecular weight ≤500, calculated octanol–water partition coefficient, log *P* ≤ 5, hydrogen bond donors ≤5, acceptors ≤10 and van der Waals bumps polar surface area (PSA) <120 Å^2^ (Muegge 2003). In the present study, all the molecular properties for all the lead compounds were estimated by using Molinspiration program (http://www.molinspiration.com/cgi-bin/properties) (Buntrock 2002). Toxicity is the second important parameter need to be considered in the analysis of lead compounds. Infact, toxicity will account the failure of majority of the lead cases. In the present study, toxicity of the lead compound examined with the help of OSIRIS program (http://www.organic-chemistry.org/prog/peo/). The program was also helpful to evaluate the drug likeliness and drug score of the lead compounds. Nearly 5300 distinct substructure fragments created by 3300 traded drugs as well as 15,000 commercially available chemicals yielding a complete list of all available fragments with the associated drug likeliness. The drug score consolidates drug-likeliness, cLogP, logS, molecular weight, and toxicity risks. It is a total value which may be used to judge the compound’s overall potential to qualify for a drug.

### Molecular docking

The docking study is immensely important to understand the bioactivity of the screened lead compounds. Initially, SMILES strings were used for constructing three dimensional structures of all the lead compounds. Subsequently, docking algorithm was performed with the help of Patch dock server (Schneidman et al. 2005). It is a molecular docking algorithm based on geometry. The energy minimized PDB coordinate file corresponds to the protein and the ligand molecule is the input parameters for the docking. This algorithm has three major stages (1) molecular shape representation (2) surface patch matching and (3) filtering and scoring. The Patch Dock services were available at http://bioinfo3d.cs.tau.ac.il/PatchDock/. The docked complexes were ranked based on the geometric matching score with target proteins. The geometric matching score of crizotinib with target proteins (native and mutant structures) were used as reference for filtering the lead compounds.

### Molecular dynamics simulation

GROMACS Package 4.5.3 implemented with Gromos 43a1 force field was utilized to perform molecular dynamics (MD) of docked complexes such as native-type ALK-crizotinib complex, mutant-type ALK-crizotinib complex, native-type ALK-CID11562217 complex and mutant-type ALK-CID11562217 complex (Hess et al. 2008; Spoel et al. 2005). The protein was solvated in cubic 0.9 nm with the help of periodic boundary conditions and the SPC water model (Meagher and Carlson 2005).This resulted in the addition of 22,269 and 23,506 water molecules to the native and mutant complex structures, respectively. PRODRG server was used to generate topology of the ligand (Schuttelkopf and Van Aalten 2004). This server uses the GROMOS force field for generating topology file and assigning atom types. Six sodium (6 Na+ ions) counter ions were added to neutralize the total charge of the system and one thousand steps of steepest descent energy minimization were carried out for the proteins. After the energy minimization step, the system was equilibrated at constant temperature and pressure. Using an atom-based cutoff of 8 Å, the non bonded list was generated. Constrains bond lengths at their equilibrium values were handled by SHAKE algorithm and the long range electrostatic interactions were handled by particle-mesh Ewald algorithm (Darden et al. 1999; Van Gunsteren and Berendsen 1977). The total simulation time was set to 20,000 ps with integration time step of 2 fs. Structural analysis was done at every picosecond and trajectories were stored in traj.trr file. For instance, root mean square deviation (RMSD) was analyzed with the help of Gromacs utilities g_rms.

## Results and discussion

### Virtual screening and bioavailability analysis

**Fig. 1:**
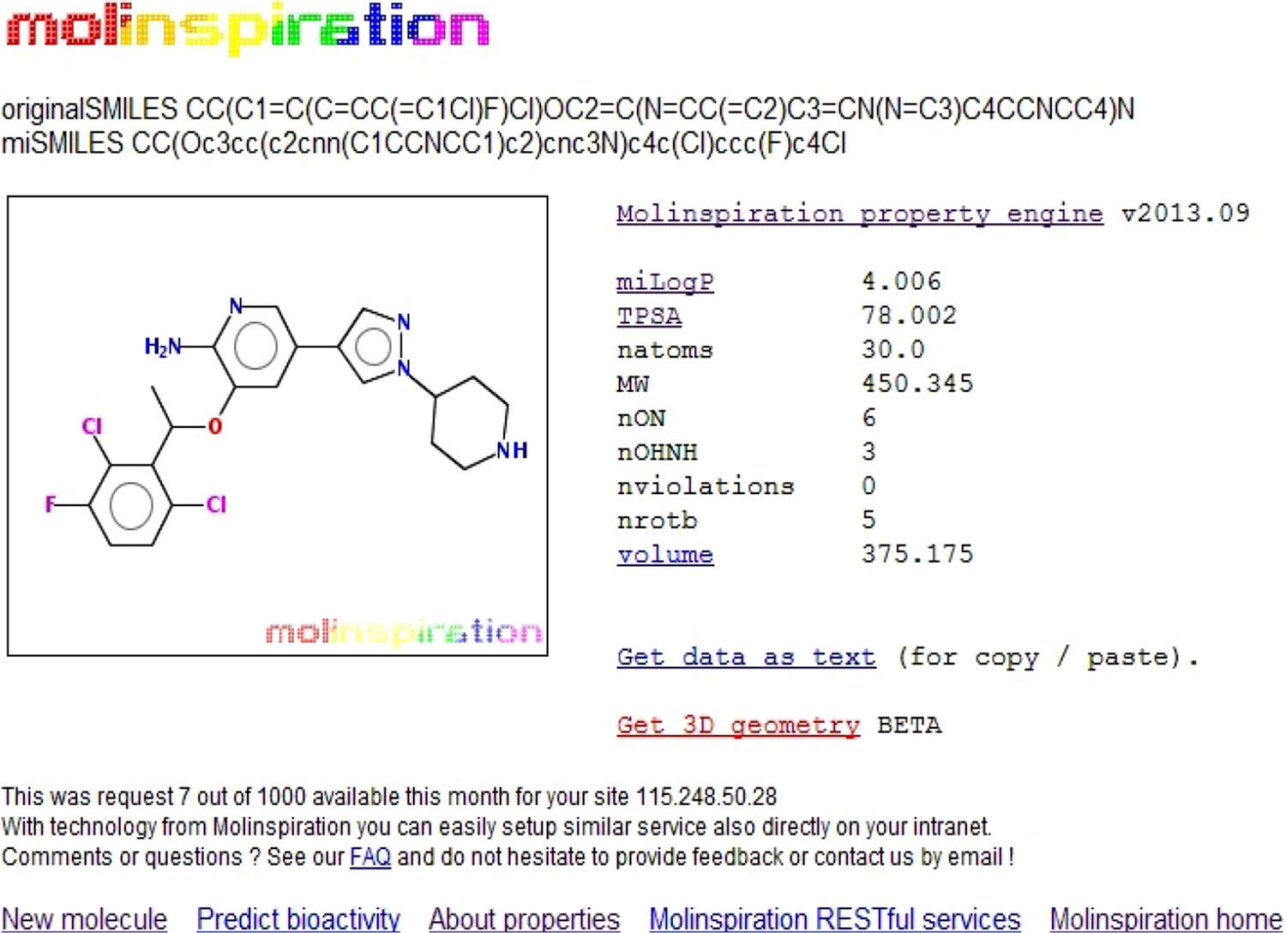
Molinspiration property explorer showing molecular properties of crizotinib

The present study initiated by extracting structurally similar compounds to crizotinib from the Pubchem database. The crizotinib was used was used as a query molecule. About 99 % similarity cutoff was maintained in the analysis. The results yield a total of 63 compounds. These compounds were utilized for our further study. Molinspiration program was used to predict the bioavailability of crizotinib and the lead compounds. Initially, crizotinib properties were calculated with the help of Molinspiration program (Fig. 1) and used as a control for screening the other lead compounds. The result is shown in Table 1. It is clear from the table that 3 compound such as CID: 11656144, CID: 11502981 and CID: 58659185 showed violations for the rule of five. The remaining 60 compounds have zero violations for the rule of five. This brings to the conclusion that bioavailability of these 60 compounds was significantly better in our dataset.

**Table 1:** Calculations of molecular properties of crizotinib and lead compound using molinspiration

S. no	Compound	miLogP	TPSA	MW	nON	nOHNH	nviolations	Volume
**1**	Crizotinib	4.006	78.002	450.345	6	3	0	375.175
**2**	**CID:11597571**	**4.006**	**78.002**	**450.345**	**6**	**3**	**0**	**375.175**
**3**	**CID: 11626560**	**4.006**	**78.002**	**450.345**	**6**	**3**	**0**	**375.175**
**4**	**CID: 53234260**	**4.006**	**78.002**	**450.345**	**6**	**3**	**0**	**375.175**
**5**	**CID:** **53234326**	**4.006**	**78.002**	**450.345**	**6**	**3**	**0**	**375.175**
**6**	**CID:** **56671814**	**4.006**	**78.002**	**450.345**	**6**	**3**	**0**	**375.175**
**7**	**CID:** **60197531**	**4.006**	**78.002**	**450.345**	**6**	**3**	**0**	**375.175**
**8**	**CID:** **60197626**	**4.006**	**78.002**	**450.345**	**6**	**3**	**0**	**375.175**
**9**	**CID:** **60198523**	**4.006**	**78.002**	**450.345**	**6**	**3**	**0**	**375.175**
**10**	**CID:** **60198524**	**4.006**	**78.002**	**450.345**	**6**	**3**	**0**	**375.175**
**11**	**CID:** **60198525**	**4.006**	**78.002**	**450.345**	**6**	**3**	**0**	**375.175**
**12**	**CID:** **60199015**	**4.006**	**78.002**	**450.345**	**6**	**3**	**0**	**375.175**
**13**	**CID:** **60199016**	**4.006**	**78.002**	**450.345**	**6**	**3**	**0**	**375.175**
**14**	**CID:** **60199073**	**4.006**	**78.002**	**450.345**	**6**	**3**	**0**	**375.175**
**15**	**CID:** **60199075**	**4.006**	**78.002**	**450.345**	**6**	**3**	**0**	**375.175**
**16**	**CID:** **60199076**	**4.006**	**78.002**	**450.345**	**6**	**3**	**0**	**375.175**
**17**	**CID:** **60199077**	**4.006**	**78.002**	**450.345**	**6**	**3**	**0**	**375.175**
**18**	**CID:** **62705017**	**4.006**	**78.002**	**450.345**	**6**	**3**	**0**	**375.175**
**19**	**CID:** **68625002**	**4.752**	**78.002**	**478.399**	**6**	**3**	**0**	**408.564**
**20**	**CID:** **54613769**	**4.006**	**78.002**	**450.345**	**6**	**3**	**0**	**375.175**
**21**	**CID:** **11662380**	**4.006**	**78.002**	**450.345**	**6**	**3**	**0**	**375.175**
**22**	**CID:** **11626823**	**4.389**	**78.002**	**464.372**	**6**	**3**	**0**	**391.977**
**23**	**CID:** **58659191**	**4.098**	**78.002**	**468.335**	**6**	**3**	**0**	**380.107**
**24**	**CID:** **44560358**	**3.643**	**78.002**	**436.318**	**6**	**3**	**0**	**358.589**
**25**	**CID: 71239831**	**4.479**	**78.002**	**490.41**	**6**	**3**	**0**	**414.441**
**26**	**CID:** **71239833**	**4.479**	**78.002**	**490.41**	**6**	**3**	**0**	**414.441**
**27**	**CID:** **71240010**	**4.479**	**78.002**	**490.41**	**6**	**3**	**0**	**414.441**
**28**	**CID:** **71240011**	**4.479**	**78.002**	**490.41**	**6**	**3**	**0**	**414.441**
**29**	**CID:** **11496366**	**4.602**	**69.213**	**464.372**	**6**	**2**	**0**	**392.118**
**30**	**CID:** **11562021**	**4.978**	**69.213**	**478.399**	**6**	**2**	**0**	**408.92**
**31**	**CID:** **11626824**	**4.602**	**69.213**	**464.372**	**6**	**2**	**0**	**392.118**
32	CID: 11656144	5.275	69.213	492.426	6	2	1	425.507
**33**	**CID:** **11598102**	**4.734**	**78.002**	**476.383**	**6**	**3**	**0**	**397.989**
**34**	**CID:** **11641497**	**3.508**	**81.24**	**479.387**	**7**	**3**	**0**	**404.735**
**35**	**CID:** **11690598**	**3.492**	**78.002**	**433.89**	**6**	**3**	**0**	**366.571**
**36**	**CID:** **68563708**	**3.492**	**78.002**	**433.89**	**6**	**3**	**0**	**366.571**
**37**	**CID:** **11562217**	**4.387**	**93.005**	**489.382**	**7**	**2**	**0**	**409.218**
**38**	**CID:** **11612136**	**4.556**	**75.209**	**451.329**	**6**	**2**	**0**	**371.758**
**39**	**CID:** **58659130**	**3.492**	**78.002**	**433.89**	**6**	**3**	**0**	**366.571**
**40**	**CID:** **11625675**	**4.921**	**65.975**	**409.292**	**5**	**2**	**0**	**339.53**
**41**	**CID:** **67084493**	**4.58**	**78.002**	**476.383**	**6**	**3**	**0**	**398.204**
**42**	**CID:** **11676204**	**3.967**	**78.002**	**424.307**	**6**	**3**	**0**	**352.147**
**43**	**CID:** **11684380**	**4.985**	**69.213**	**478.399**	**6**	**2**	**0**	**408.92**
**44**	**CID:** **58659192**	**4.825**	**78.002**	**494.373**	**6**	**3**	**0**	**402.92**
**45**	**CID:** **59599446**	**3.445**	**98.230**	**480.371**	**7**	**4**	**0**	**399.671**
**46**	**CID:** **11503318**	**4.357**	**78.002**	**450.345**	**6**	**3**	**0**	**375.175**
**47**	**CID:** **11510387**	**4.086**	**78.002**	**436.318**	**6**	**3**	**0**	**358.374**
**48**	**CID:** **11568619**	**4.357**	**78.002**	**450.345**	**6**	**3**	**0**	**375.175**
**49**	**CID:** **11575401**	**3.816**	**78.002**	**422.291**	**6**	**3**	**0**	**341.572**
**50**	**CID:** **11647760**	**4.086**	**78.002**	**436.318**	**6**	**3**	**0**	**358.374**
**51**	**CID:** **58659136**	**4.086**	**78.002**	**436.318**	**6**	**3**	**0**	**358.374**
**52**	**CID:** **58659189**	**4.291**	**78.002**	**446.382**	**6**	**3**	**0**	**386.805**
**53**	**CID:** **72986690**	**4.357**	**78.002**	**450.345**	**6**	**3**	**0**	**375.175**
**54**	CID: 11502981	5.581	65.975	435.33	5	2	1	362.773
**55**	**CID:** **11676140**	**4.842**	**65.975**	**421.303**	**5**	**2**	**0**	**345.971**
**56**	**CID:** **58659141**	**4.939**	**75.209**	**465.356**	**6**	**2**	**0**	**388.56**
**57**	**CID:** **11705849**	**4.978**	**69.213**	**490.41**	**6**	**2**	**0**	**414.932**
**58**	**CID:** **11719356**	**3.956**	**78.002**	**450.345**	**6**	**3**	**0**	**375.175**
**59**	**CID:** **11647759**	**4.199**	**78.002**	**436.318**	**6**	**3**	**0**	**358.374**
**60**	**CID:** **21110753**	**4.058**	**78.447**	**480.371**	**7**	**2**	**0**	**401.318**
**61**	CID: 58659185	5.304	65.975	423.319	5	2	1	356.331
62	**CID:** **21110757**	**4.182**	**65.975**	**381.238**	**5**	**2**	**0**	**306.141**
**63**	**CID:** **73386634**	**4.182**	**65.975**	**381.238**	**5**	**2**	**0**	**306.141**
**64**	**CID:** **11647795**	**4.285**	**75.209**	**437.302**	**6**	**2**	**0**	**354.956**

Bold indicates ADME screened compounds based on Lipinsiki rule of 5

It is bare that for passing oral bioavailability criteria, number of rotatable bond should be <10 (Oprea 2000). Therefore, we have made the further refinement of these hits by restricting the number of rotatable bonds to 10. The result is presented in Table 2. It is clear from the Table 2 that almost all the 60 compounds screened from the ADME analysis possess reasonable number of rotatable bonds (<10). This result indicates that these compounds may have the potential to become a lead compound. However, toxicity is also one of the important issue could be addressed for all the lead compounds before its selection.

**Table 2:** Details of number of rotatable bonds

S. no	Compound	nrotb
1	Crizotinib	5
2	CID: 11597571	5
3	CID: 11626560	5
4	CID: 53234260	5
5	CID: 53234326	5
6	CID: 56671814	5
7	CID: 60197531	5
8	CID: 60197626	5
9	CID: 60198523	5
10	CID: 60198524	5
11	CID: 60198525	5
12	CID: 60199015	5
13	CID: 60199016	5
14	CID: 60199073	5
15	CID: 60199075	5
16	CID: 60199076	5
17	CID: 60199077	5
18	CID: 62705017	5
19	CID: 68625002	6
20	CID: 54613769	5
21	CID: 11662380	5
22	CID: 11626823	6
23	CID: 58659191	5
24	CID: 44560358	5
25	CID: 71239831	5
26	CID: 71239833	5
27	CID: 71240010	5
28	CID: 71240011	5
29	CID: 11496366	5
30	CID: 11562021	6
31	CID: 11626824	5
32	CID: 11598102	5
33	CID: 11641497	7
34	CID: 11690598	5
35	CID: 68563708	5
36	CID: 11562217	5
37	CID: 11612136	5
38	CID: 58659130	5
39	CID: 11625675	5
40	CID: 67084493	6
41	CID: 11676204	7
42	CID: 11684380	6
43	CID: 58659192	5
44	CID: 58659228	5
45	CID: 11503318	6
46	CID: 11510387	5
47	CID: 11568619	6
48	CID: 11575401	5
49	CID: 11647760	5
50	CID: 58659136	5
51	CID: 58659189	5
52	CID: 72986690	6
53	CID: 11676140	5
54	CID: 58659141	6
55	CID: 11705849	5
56	CID: 11719356	5
57	CID: 11647759	6
58	CID: 21110753	7
59	CID: 21110757	4
60	CID: 73386634	4
61	CID: 11647795	5

Number of rotatable bonds <10

### Toxicity analysis

The primary objective behind the failure of the majority of compounds in drug discovery process is the issues related to pharmacokinetics and toxicity. In the present investigation, these issues were addressed with the help of OSIRIS property explorer program. The pharmacokinetic property of a lead compound can be investigated by utilizing the parameters such as clogP and logS. The result is shown in Table 3. clogP is an entrenched measure of the compound’s hydrophilicity. The high log *P* values may cause poor retention because of the compound’s low hydrophilicity. It has been demonstrated that for compounds to have a reasonable probability of being well absorbed, their log *P* value must not be greater than 5.0. It is clear from the table that log *P* values of all the 60 compounds found to be in the acceptable criteria.

**Table 3:** Toxicity risks and physicochemical properties of crizotinib and virtual compounds predicted by OSIRIS property explorer

S. no	Compound ID	Mutagenic	Tumorigenic	Reproductive effective	cLogP	Solubility	Drug likeness	Drug score
1	Crizotinib	No	No	No	3.54	−5.26	3.12	0.52
2	CID: 11597571	No	No	No	3.54	−5.26	3.12	0.52
3	CID: 11626560	No	No	No	3.54	−5.26	3.12	0.52
4	CID: 53234260	No	No	No	3.54	−5.26	3.12	0.52
5	CID: 53234326	No	No	No	3.54	−5.26	3.12	0.52
6	CID: 56671814	No	No	No	3.54	−5.26	3.12	0.52
7	CID: 60197531	No	No	No	3.54	−5.26	3.12	0.52
8	CID: 60197626	No	No	No	3.54	−5.26	3.12	0.52
9	CID: 60198523	No	No	No	3.54	−5.26	3.12	0.52
10	CID: 60198524	No	No	No	3.54	−5.26	3.12	0.52
11	CID: 60198525	No	No	No	3.54	−5.26	3.12	0.52
12	CID: 60199015	No	No	No	3.54	−5.26	3.12	0.52
13	CID: 60199016	No	No	No	3.54	−5.26	3.12	0.52
14	CID: 60199073	No	No	No	3.54	−5.26	3.12	0.52
15	CID: 60199075	No	No	No	3.54	−5.26	3.12	0.52
16	CID: 60199076	No	No	No	3.54	−5.26	3.12	0.52
17	CID: 60199077	No	No	No	3.54	−5.26	3.12	0.52
18	CID: 62705017	No	No	No	3.54	−5.26	3.12	0.52
19	CID: 68625002	No	No	No	3.78	−5.69	3.68	0.46
20	CID: 54613769	No	No	No	3.54	−5.26	3.22	0.53
21	CID: 11662380	No	No	Yes	3.54	−5.26	2.78	0.42
22	CID: 11626823	No	No	No	3.29	−5.78	3.45	0.48
23	CID: 58659191	No	Yes	No	3.64	−5.58	3.17	0.29
24	CID: 44560358	No	No	No	3.25	−5.19	2.42	0.54
25	CID: 71239831	No	No	No	4.19	−5.96	1.79	0.38
26	CID: 71239833	No	No	No	4.19	−5.96	1.45	0.37
27	CID: 71240010	No	No	No	4.19	−5.96	1.79	0.38
28	CID: 71240011	No	No	No	4.19	−5.96	1.79	0.38
29	CID: 11496366	No	No	No	3.79	−4.90	7.62	0.54
30	CID: 11562021	No	No	No	4.2	−5.22	7.51	0.48
31	CID: 11626824	No	No	No	3.79	−4.90	7.62	0.54
32	CID: 11598102	No	No	No	3.89	−6.11	2.11	0.41
33	CID: 11641497	No	No	No	2.38	−4.53	4.34	0.49
34	CID: 11690598	No	No	No	3.03	−4.84	3.12	0.6
35	CID: 68563708	No	No	No	3.03	−4.84	3.12	0.6
36	CID: 11562217	No	No	No	3.44	−5.35	2.82	0.29
37	CID: 11612136	No	No	No	3.68	−5.4	−0.93	0.33
38	CID: 58659130	No	No	No	3.03	−4.84	3.22	0.60
39	CID: 11625675	No	No	No	3.75	−5.39	2.56	0.53
40	CID: 67084493	No	No	No	4.04	−6.15	1.21	0.37
41	CID: 11676204	No	No	No	2.28	−4.96	3.76	0.62
42	CID: 11684380	No	No	No	3.55	−5.42	7.62	0.54
43	CID: 58659192	No	Yes	No	4	−6.42	2.17	0.22
44	CID: 59599446	No	No	No	2.47	−5.33	4.07	0.53
45	CID: 11503318	No	No	No	3.01	−5.73	0.63	0.42
46	CID: 11510387	No	No	No	3.30	−6.39	3.37	0.47
47	CID: 11568619	No	No	No	3.01	−5.73	0.63	0.42
48	CID: 11575401	No	No	No	2.85	−4.72	3.34	0.63
49	CID: 11647760	No	No	No	3.20	−4.99	3.81	0.58
50	CID: 58659136	No	No	No	3.20	−4.99	3.81	0.48
51	CID: 58659189	Yes	No	No	3.78	−5.29	4.46	0.31
52	CID: 72986690	No	No	No	3.01	−5.73	0.63	0.42
53	CID: 11676140	No	No	No	4.16	−5.75	1.66	0.44
54	CID: 58659141	No	No	No	3.44	−5.91	−0.32	0.33
55	CID: 11705849	No	No	No	4.15	−5.74	2.96	0.42
56	CID: 11719356	No	No	No	3.63	−4.96	2.31	0.53
57	CID: 11647759	No	No	No	2.61	−5.24	3.45	0.57
58	CID: 21110753	No	No	No	2.52	−4.67	3.67	0.59
59	CID: 21110757	No	No	No	3.00	−5.47	2.35	0.56
60	CID: 73386634	No	No	No	3.00	−5.47	2.35	0.56
61	CID: 11647795	No	No	No	3.34	−5.13	0.85	0.48

Drug solubility normally affects the absorption and distribution characteristics of a compound. Infact, insufficient solubility of drug can lead to poor absorption (Lipinski et al. 1997). Our evaluated log *S* worth is a unit stripped logarithm (base 10) of a compound’s dissolvability measured in mol/liter. There are more than 80 % of the drugs available in the market have an (expected) log *S* value greater than −4. It is clear from the Table 3 that the solubility of the 60 lead compounds was found in the comparable zone with that of standard drugs to fulfill the requirements of solubility and this could be regarded as a candidate drug for oral absorption.

### Drug likeness

The drug likeliness is imperative parameter because drug like molecules exhibit favorable absorption, distribution, metabolism, excretion, toxicological (ADMET) parameters (Tetko 2005). In this study, Osiris program was utilized to calculate the drug-likeness of crizotinib and other virtually screened compounds (Sander 2001). It is worth stressing that the drug likeness value of 60 lead compounds was found to be in acceptable criteria.

### Drug score and toxicity

The information assessed in Table 3 shows that the 57 lead compounds should be non-mutagenic and non-tumorigenic impacts when run through the mutagenicity assessment system comparable with standard drugs used. The compounds such as CID: 11662380, CID: 58659189, CID: 58659191, and CID: 58659192 failed to pass through the Osiris program and showed mutagenic and tumorigenic effects. We have also analyzed the overall drug score (DS) for all the lead compounds and compared with that of crizotinib. The score consolidates drug- likeness, miLogP, logS, molecular weight, and toxicity risks. The DS score could also be an important parameter to judge the compound’s potential to meet all requirements to qualify for a drug. The result is demonstrated in Table 3. The reported lead compounds demonstrated moderate to good DS as compared with standard drug crizotinib. In our dataset, 17 lead compounds showed similar drug score as that of crizotinib. About five compounds such as CID: 11690598, CID: 68563708, CID: 58659130, CID: 11676204 and CID: 11575401 showed a drug score of 0.6 and above. Therefore, further examination was carried out with 57 compounds.

### Molecular docking

Molecular docking program was employed to find out the binding affinity of lead compounds with the target protein. Docking analysis was performed twice to eliminate the false positive. The docking results are shown in Table 4. The docking score of native-type ALK-crizotinib complex was found to be 5312 and for the mutant-type ALK-crizotinib complex was found to be 4602. The lesser docking score of mutant complex clearly indicates that double mutation (L1196M and G1269A) significantly affects the binding of crizotinib with the ALK structures. It is believed that a potential lead compound is the one should have higher docking scoring than the existing drug molecule, crizotinib. Therefore, we have examined docking score for all the 57 hits both with the native type and with mutant type ALK systems. 16 hits showed higher docking score only with mutant type ALK than native type ALK and 17 more hits from our dataset showed similar dock score to that of crizotinib. Most importantly, 10 hits from our dataset showed higher score both in the native type as well as with mutant type. For instance, CID 11562217 molecule showed the highest docking score among the 10 hits in our data set. The docking score of native-type ALK-CID 11562217 complex was found to be 5662 and for the mutant-type ALK-CID 11562217 complex was found to be 5908. This result indicates that CID 11562217 has a better binding affinity not only with the native type but also with mutant ALK as compared to the crizotinib.

**Table 4:** Docking score of the crizotinib and lead compounds obtained from PubChem database against the target structure

S. no	Compound ID	Score	
		2XP2	4ANS
1	Crizotinib	5312	5226
2	CID: 11597571	5312	5226
3	CID: 11626560	5312	5226
4	CID: 53234260	5312	5226
5	CID: 53234326	5312	5226
6	CID: 56671814	5312	5226
7	CID: 60197531	5312	5226
8	CID: 60197626	5312	5226
9	CID: 60198523	5312	5226
10	CID: 60198524	5312	5226
11	CID: 60198525	5312	5226
12	CID: 60199015	5312	5226
13	CID: 60199016	5312	5226
14	CID: 60199073	5312	5226
15	CID: 60199075	5312	5226
16	CID: 60199076	5312	5226
17	CID: 60199077	5312	5226
18	CID: 62705017	5312	5226
19	CID: 68625002	5200	5342
20	CID: 54613769	5298	5308
21	CID: 11626823	5048	5226
22	CID: 44560358	5012	5386
**23**	**CID:** **71239831**	**5440**	**5776**
**24**	**CID:** **71239833**	**5440**	**5776**
**25**	**CID:** **71240010**	**5426**	**5504**
**26**	**CID:** **71240011**	**5426**	**5504**
**27**	**CID:** **11496366**	**5412**	**5420**
**28**	**CID:** **11562021**	**5510**	**5492**
**29**	**CID:** **11626824**	**5412**	**5420**
30	CID: 11598102	5292	5294
31	CID: 11641497	5450	5138
32	CID: 11690598	4906	5138
33	CID: 68563708	4906	5138
**34**	**CID:** **11562217**	**5662**	**5908**
35	CID: 11612136	5144	5032
36	CID: 58659130	5108	5294
37	CID: 11625675	4746	5052
38	CID: 67084493	4950	5334
39	CID: 11676204	4964	4962
40	CID: 11684380	4964	5424
**41**	**CID:** **59599446**	**5434**	**5704**
42	CID: 11503318	5110	5138
43	CID: 11510387	5124	5372
44	CID: 11568619	5110	5138
45	CID: 11575401	4886	4826
46	CID: 11647760	5124	5372
47	CID: 58659136	5124	5372
48	CID: 72986690	5110	5138
49	CID: 11676140	4906	5484
50	CID: 58659141	5118	5278
51	CID: 11705849	5186	5370
52	CID: 11719356	5040	5238
53	CID: 11647759	5026	5118
**54**	**CID:** **21110753**	**5390**	**5526**
55	CID: 21110757	4408	4604
56	CID: 73386634	4408	4604
57	CID: 11647795	5268	5212

Bold indicates the lead compounds showed higher binding score than crizotinib

**Fig. 2:**
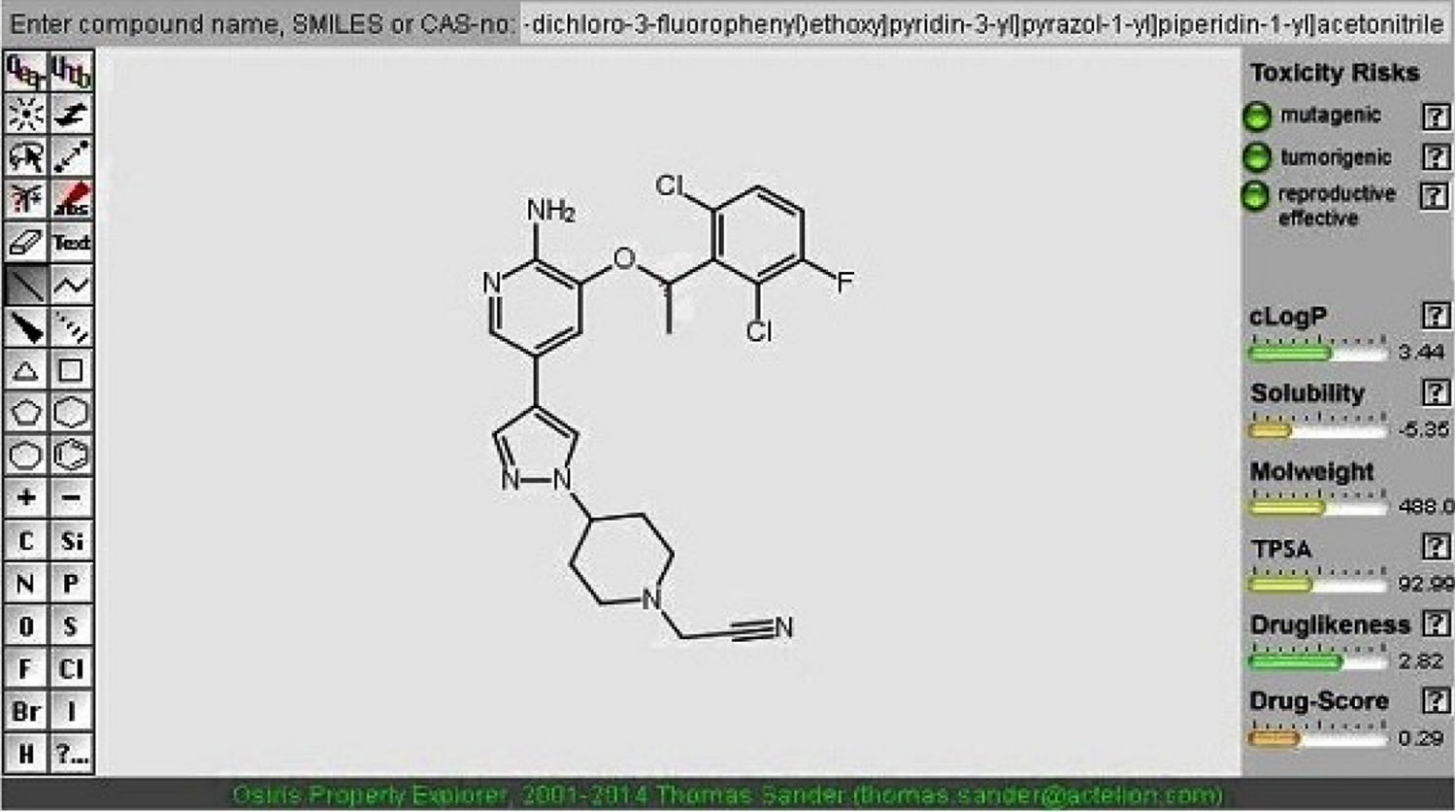
Osiris property explorer showing drug-likeliness properties of CID11562217

**Fig. 3:**
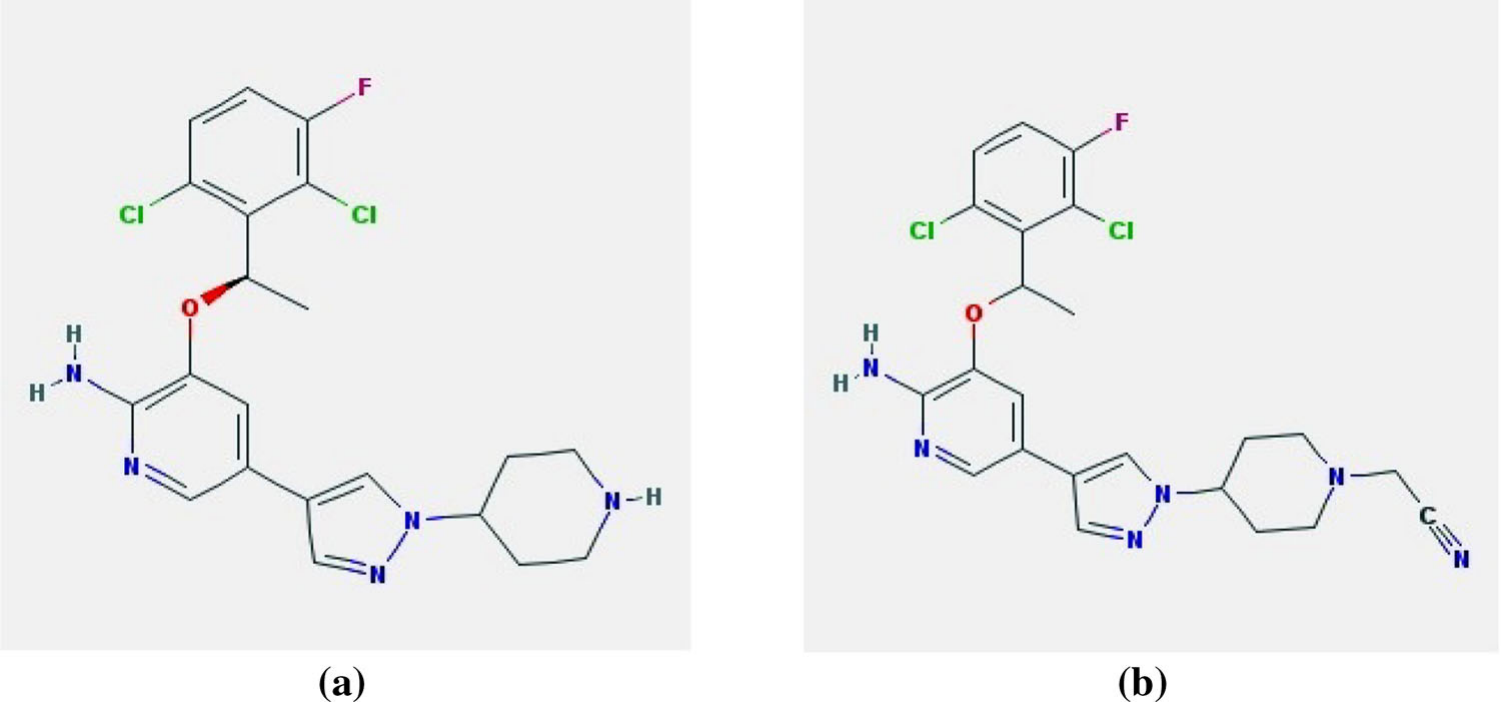
Structure comparison between (**a**) crizotinib and (**b**) CID11562217

It is also to be noted that the pharmacokinetic and pharmacodynamic investigation of CID 11562217 indicated better results than the other lead compounds explored in our study (Fig. 2). The two dimensional structure of crizotinib was compared with CID 11562217 to get the structural attributes and the result is demonstrated in Fig. 3. It demonstrates that CID11562217 is a nitrile enhanced crizotinib. It is worth stressing that nitrile compounds with cyanide functional group could possess potential anti-tumor effects (US Patent 20060128724). The literature evidence also highlights that our lead molecule has kinase inhibiting effects. Further, the cyano-containing analogues were able to produce DNA–DNA cross-linking. The reduced DNA cross-linking was paralleled by a similar reduction in cytotoxicity indicating a relationship between cross-linking and anti-tumor effect (Jesson et al. 1987). Therefore, further validation of CID 11562217 compound was done with the help of molecular dynamics simulation study.

### Molecular dynamics simulation

**Fig. 4:**
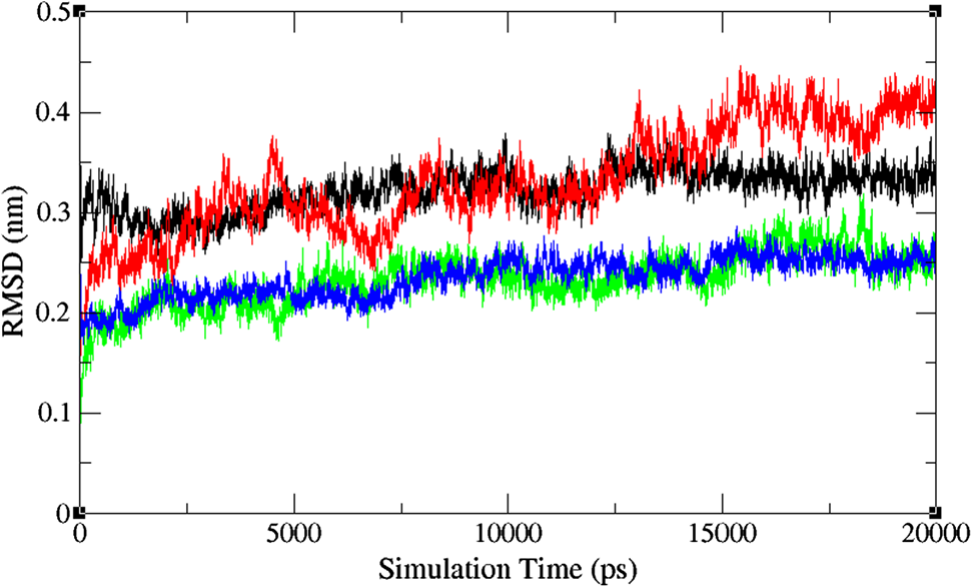
Root mean square deviations correspond to native-type ALK-crizotinib complex (*black*), mutant-type ALK-crizotinib complex (*red*), native-type ALK-CID11562217 complex (*green*) and mutant-type ALK-CID11562217 complex (*blue*) along the MD simulation at 300 K

Molecular dynamics simulation study was carried out with the help of GROMACS package 4.5.3 to explore the stability of the complex structures. In particular, the parameter, RMSD, was examined from the trajectory file and used for analyzing the complex stability. RMSD investigation can give a thought of how much the three-dimensional structure has deviated over the time. The result is shown in Fig. 4. Native type ALK-crizotinib complex structure acquired ~0.34 nm at 1000 ps during the simulations, while mutant type ALK-crizotinib complex structure acquired ~0.28 nm of backbone RMSD at 1000 ps. On the other hand, native-type ALK-CID11562217 structure acquired ~0.18 nm of backbone RMSD while mutant-type ALK-CID11562217 complex structure acquired ~0.22 nm of backbone RMSD at 1000 ps. Between a period of 2000–5000 ps, native type ALK-crizotinib complex structure maintains a RMSD value of ~0.30 nm whereas mutant type ALK-crizotinib complex structure showed a deviation from ~0.25 to ~0.36 nm. In the virtual complex, native-type ALK-CID11562217 structure showed a RMSD value between ~0.18 and ~0.20 nm and mutant type ALK-CID11562217 complex structure maintains a RMSD value of ~0.24 nm. From the period of 5000–10,000 ps, native-type ALK-crizotinib complex structure maintains a RMSD value of ~0.34 nm while, mutant type ALK-crizotinib complex has deviated from ~0.32 to ~0.36 nm. On the contrary, native-type ALK-CID11562217 complex structure maintains a RMSD value of ~0.25 nm while mutant type ALK-CID11562217 complex structure maintains a RMSD value of ~0.20 to ~0.24 nm. From the beginning of 11,000 ps to the end of 15,000 ps, mutant type ALK-crizotinib complex structure showed higher deviation and attains a RMSD value of ~0.44 nm while native-type ALK-crizotinib complex structure maintains a RMSD value of ~0.23 nm. Mutant type ALK-CID11562217 complex structure maintains a RMSD value of ~0.25 nm in this simulation period. Between a period of 16,000–19,000 ps, native type ALK-crizotinib complex structure maintains a RMSD value of ~0.35 nm whereas mutant type ALK-crizotinib complex structure showed a deviation from ~0.43 to ~0.45 nm. For instance, native-type ALK-CID11562217 structure showed a RMSD value of ~0.25 nm and mutant type ALK-CID11562217 complex structure maintains a RMSD value of ~0.22 nm. At the end of 20,000 ps the mutant type ALK-crizotinib complex structure attained RMSD of ~0.40 nm and native type ALK-crizotinib complex structure attained RMSD of ~0.35 nm. This clearly indicates that ALK double mutation disturb the structural stability and also its function. It is worth stressing that native and mutant type ALK-CID 11562217 able to maintain a RMSD of ~0.24 nm. Overall, significant difference in RMSD value observed between the crizotinib and CID 11562217 complex system. The lesser RMSD value of CID 11562217 complex demonstrates the stable binding of CID 11562217 with both native and mutant type ALK structures.

## Conclusion

In the present investigation, we have addressed the crizotinib resistance in NSCLC with the help of virtual screening approach. CID 11562217 was discovered to be more drug like as it productively passed through the parameters of pharmacokinetics and toxicity. Docking study demonstrated that CID 11562217 has the highest binding affinity not only with native type ALK but also with the mutant type ALK system among the lead compounds screened from the Pubchem database. RMSD data obtained from molecular dynamic simulation revealed structural stability of the ALK-CID11562217 complex structure. It is worth stressing that our results correlate well with available experimental evidences. Of note, the available data suggests that pyrazole-substituted aminoheteroaryl compounds have potential anti-tumor effects. We hope that the findings reported here might give helpful signs to design powerful drugs against drug resistant lung cancer types.
